# Access to Physical Activity and Sport and the Effects of Isolation and Cordon Sanitaire During COVID-19 for People With Disabilities in Scotland and Canada

**DOI:** 10.3389/fspor.2020.594501

**Published:** 2020-12-23

**Authors:** Denise Kamyuka, Liz Carlin, Gayle McPherson, Laura Misener

**Affiliations:** ^1^School of Kinesiology, Western University, London, ON, Canada; ^2^School of Health and Life Sciences, University of West Scotland, Paisley, United Kingdom; ^3^School of Business and Creative Industries, University of West Scotland, Paisley, United Kingdom

**Keywords:** disability, sport, physical activity, COVID-19, isolation, inequalities

## Abstract

People with a disability are more at risk of experiencing inequalities in relation to sporting and physical activity opportunities, COVID-19 and the resulting restrictions stand to exacerbate these inequalities. This research directly contributes toward the World Health Organization, global research roadmap priority to explore “the impact of restrictive public health measures (e.g., quarantine, isolation, cordon sanitaire).” Social loneliness and social isolation have a significant impact on the health and wellbeing of individuals, therefore, it is imperative to gain an understanding of the effects of self-isolation and shielding during Covid-19 for people with disabilities to help those in policy and agency positions address these issues. This research utilized a qualitative approach, to speak with people with a disability, athletes and non-athletes and those in a position of management and policy making. Six online focus groups, with people participating in sport and physical activity, using live captioning, chat functions, and an online written discussion forum; through Project Echo website as part of a wider study on sport and accessibility were utilized. The study also drew on interviews and one of the focus groups was with senior policy makers and regional managers responsible for disability sport in Scotland.

## Introduction

The aim of this research is to understand how People with a Disability (PwD)^1^ have been impacted by Covid-19, specifically in relation to their ability to participate in sport or physical activity. It is known that a decrease in physical activity is a risk factor for secondary non-communicable diseases (van Schijndel-Speet et al., 2014). It is therefore pertinent that groups at a higher risk of acquiring secondary chronic conditions are provided with adequate opportunities to participate in physical activity. Social loneliness and social isolation have a significant impact on the health and well-being of individuals (Macdonald et al., 2018) therefore it is imperative to gain an understanding of the effects of self-isolation and shielding during Covid-19 for PwD. Vice versa, it is also important to explore if reduced access to physical activity could exacerbate the challenges of social isolation and loneliness for groups such as PwD, during lockdown. This impetus for this research stems from the need to investigate the impact of reductionist measures on PwD, a population that already faced recognized socio-economic challenges before Covid-19. Researching the impact of these measures on existing pressures, combined with new ways of exercising and engaging in sport and physical activity is brought about as a result of slowly emerging from lockdown represents a unique and valuable opportunity to understand the needs of PwD and to build connectedness and resilience for those who find themselves, excluded further from society due to Covid-19, and resultant government responses.

## Rationale

There are concerns that governments will exacerbate the marginalization of PwDs, by neglecting to ensure that Covid-19 responses are inclusive and accessible for all. A news release by (UN News, 2020) raised awareness to the following key considerations for avoiding increased disparity in treatment of PwD included: safeguarding access to healthcare as a priority, disseminating Covid-19 related information through accessible mediums, training of essential workers on non-discriminatory treatment of PwD, and providing support to ensure that the economic statuses of PwD do not deteriorate any further.

The Canadian government's last announcement on PwD and the Covid-19 response, addressed the concerns of the UN. The government published recommendations and considerations for organizations and people working with PwD. These considerations reiterated the UN's concerns but also included: anticipated difficulties with the use of face masks and visors, provisions for caregivers, and the need for gender sensitive response measures (Canada.Ca, 2020). The government, however, did not offer any formal policy for the implementation of these considerations. Instead in response to the pandemic and its economic impact on PwD, the federal and Ontario government in Canada offered PwD up to $600 CAD in financial support. They provided up to $15 million CAD in funding to support the employment of PwD and $1.8 million CAD to fund the technology upgrades and projects designed to make work from home systems more accessible (Prime Minister of Canada, 2020).

Within the UK, the COVID-19—Framework for Decision Making (2020) was introduced into legislation to provide emergency powers to respond to the Coronavirus pandemic including the provision of support for PwD. Specifically, within the devolved Scottish government, work continues with key stakeholders such as Disability Equality Scotland, Inclusion Scotland and Glasgow Disability Alliance to support the provision of essential services and to maintain communication from its members on key issues facing PwD including the wearing of face masks. Additionally, £350 million community funding was provided to support the access of food to those shielding including PwD with the Covid-19 Transition Fund also assisting organizations as they manage the evolving situation and the changing needs of their members in a way that will ensure sustainability.

Monetary relief packages and investments that bolster work at home accessibility features, are paramount in the support for PwD. However, we believe that access to physical activity can contribute significantly to providing holistic support during the pandemic, amidst lockdown restrictions.

### Aims and Objectives

This research aimed to gain an understanding of the experience and perceptions of people with disabilities, with regards to the Covid-19 lockdown in Canada and Scotland. We aimed to understand how the associated closures of parks and open spaces have impacted their ability to be physically active. In addition, we aimed to understand the perceptions of those in management or policy positions who are trying to aid accessibility and participation in disability sport. The research examines both the reduction in access to facilities, venues, and services, such as personal care and looks at how engagement from sport disability groups, charities, and family members have acted as a lifeline for social contact, physical engagement and for those who are athletes' online access to peers. To do so, we draw upon the theoretical understandings of the biopsychosocial model of disability focusing on how structural and economic barriers have led to further isolation for PwD who were already marginalized from society. Thus, we address this by examining how PwD have been affected by the social, cultural, and economic conditions to access physical activity brought about by Covid-19 and the policy responses aimed at safeguarding the population.

### Literature Review

The literature draws on contemporary debates emerging from academic and gray literature centering around the biopsychosocial model of disability and the United Nations (UN) Convention of Human Rights that every individual has a right to access leisure and recreation (UN, 2006). Furthermore, we draw from literature on loneliness, social exclusion and mental health. Previous work has highlighted that often an individuals' disability is only one form of oppression that they are experiencing, and they are only prevented from doing so because of other social, cultural or political constraints (Misener et al., 2019).

#### Social Model of Disability

The framework of this paper is based on the social model of disability (SMD). The model borrows claims from the Union of the Physically impaired Against Segregation (UPIAS) in 1975, that disability is not merely a medicalized condition but rather an imposition of what limitations society perceives a PwD would face due to their impairment (Shakespeare, 2006). In the fight for equality, it has been argued that this misinterpretation of disability has resulted in the exclusion of PwD from economic and recreation participation in society. Rather than insisting that PwD adapt to society, the social model of disability, scrutinizes social structures, and instead addresses how society itself can adapt to people with an impairment (Hughes, 2000).

The SDM however, is not free from criticism. In many ways, it fails to take into account the heterogeneity of impairment and how this varies according to the lived experiences of PwD. For a more tempered approach, this study included the Biopsychosocial Model of disability (BMD). This model is predicated on the SDM, with the additional feature of accounting for the subjective experiences of PwD. The BMD is based on psychological variables as well as biological variables (Waddell et al., 2002). According to George and Engel (1980) this interaction between psychological and medical must be explored within the myriad of social systems, this thus provides a premise for this study. In addition to the social variables, this model allows us to incorporate the psychological and emotional variables that contribute to or precipitate from an inability to participate in physical activity.

#### Loneliness and People With Disabilities

According to cognitive theory, loneliness is experienced by people who perceive a deficit in their desired amount of meaningful social interaction (Perlman and Peplau, 1982). It is important to highlight that the cognitive characteristics of loneliness are dependent on how an individual perceives their social relationships as inadequate or of low quality. Loneliness, which is synonymous with social isolation, is subjectively measured against the quality of relationships an individual has had in the past or against the quality of relationships the individual perceives others to have (de Jong Gierveld, 1998; Hawkley and Cacioppo, 2010). In 2017, 1.3 million PwD over the age of 15 (21%), reported living alone, thus justifying a concern for the onset of loneliness experienced by this population during lockdown (Statistics Canada, 2020).

There is a corpus of longitudinal research that identifies both chronic and acute loneliness as a risk factor to increased morbidity and mortality (Hawkley and Cacioppo, 2010; Holt-Lunstad et al., 2015; Leigh-Hunt et al., 2017). Loneliness is shown to diminish individuals' abilities to self-regulate thoughts, feelings, and behavior. This often results in a negative effect which could reduce motivation for physical activity (Hawkley and Cacioppo, 2010; Holt-Lunstad et al., 2015; Leigh-Hunt et al., 2017). Acute or “Situational” loneliness is described by Shiovitz-Ezra and Ayalon (2010, p. 456), as a short period of increased stress caused by a loss or reduction in quality social relationships. Acute loneliness experienced by PwD during the Covid-19 pandemic and subsequent lockdowns, could have catalytic effects on the deterioration of their physiological conditions, particularly for people with progressive conditions. Hawkley and Cacioppo (2010), propose that loneliness is affiliated with perpetual pessimism, feeling unsafe, anxiety, and low self-esteem. This results in an exhibition of paranoid and overly cautious behavior toward the environment and people outside of the PwDs' intimate circles. This perceived threat is often projected onto others and elicits a negative reaction from people in society, thus perpetuating the deviant identity of PwD. Research has shown that, in many cases, PwD relate loneliness to their bodily and social differences (Tarvainen, 2020). Research has in fact indicated that physical activity can play an important role in reducing social isolation, particularly for groups more at risk of experiencing isolation and loneliness (Schrempft et al., 2019).

#### Negative Social Perceptions on Disability

The “deviant” identity is a socially imposed perspective that often contributes to the exclusion of PwD from society (Low, 1996, p. 236). We turn to ableism literature to provide us with the vocabulary to dissect social norms that inherently create barriers to inclusion and participation. Ableism is also predicated on the Social Model of Disability, however, attributing discrimination in favor of people without disability. Ableism in essence addresses society's tendency to conveniently accommodate the non-disabled population. Campbell (2009) introduced the term internalized ableism to describe the self-inflicted oppression imposed by PwD who prefer to shy away from their impairment, either as a result of self-loathing or a need to blend in with the able bodied. Campell draws from critical race studies to describe the concept of “passing” (p. 26), in which a person with a disability works to mask their impairment or avoid questions about their disability. In this they hope to create a space where they are “not disturbing the peace, by containing the matter that is potentially out of place” (p. 26).

Brittain et al. (2020), propose a model that identifies ableism as a regulating mechanism to self-determination and consequently participation in physical activity. Self-determination speaks to one's ability to self-motivate. Brittain et al. (2020) argue that the self-motivation to participate in physical activity is curtailed by ableism and its effects on autonomy, competence, and relatedness. Under the lens of ableism, there is a misconception that PwD have little autonomy, worse yet, they are increasingly robbed of their autonomy by the various barriers to participation that exist as a result of this misconception. The authors (Brittain et al., 2020) believe that PwD's perceived lack of competence with physical activity is explicated by internal ableism, which further impedes self-motivation. Lastly, the ability to relate to, and through, physical activity is a much-celebrated (in normative culture) tenet of sport and physical activity, however, external ableism impacts on PwD's “relatedness” to physical activity (Brittain et al., 2020, p. 220). The ability to relate facilitates the building of social networks and thus social capital. An inability to relate therefore limits one's sense of belonging and creates constraints to participation. It is therefore apparent that in order to dismantle barriers informed by ableism, disability identity needs to be recognized as different rather than being ignored, made invisible, or homogenized (Loja et al., 2013). This reaffirms the need to go beyond the Social Model of Disability, toward a tempered perspective that involves a Biopsychosocial Model of Disability. The biopsychosocial model also allows us to incorporate the heterogenic responses and experience with the Covid-19 lockdown measure, attributed to various disabilities and levels of disability represented in our research.

Exclusion from sport and physical activity for PwD, is best evaluated in terms of experiences with constraints to participation (Darcy et al., 2017). The most common constraints experienced are associated with a lack of financial resources, transportation, sports coaches/instructors, and accessibility features (Darcy et al., 2017). In many developing countries, negative social perceptions of PwD presented the greatest constraint on participation. With regards to participation in outdoor recreation PwD face the added constraint of not having a person or group to accompany them (Burns and Graefe, 2007).

Studies however show that constraints do not necessarily equate to a decrease in participation (Darcy et al., 2017). So, in addition to understanding the perceptions toward participating in physical activity, this paper looks at some mechanisms employed to help overcome the constraints to participation in physical activity during lockdown periods.

#### Physical Activity and Mental Health

Research exploring the relationship between physical activity and mental health, allude to a feedback mechanism that connects self-determination theory to the positive effects of participating in physical activity, namely the social connectedness or “relatedness.” To further explain the loop, self-determination provides the motivation to participate and experience meaningful social connections, social connections augment control over positive cognitive behavior which in turn promotes self-determination, and the loop continues (Burke et al., 2006; Graham et al., 2008). The literature on the psychological effects of barriers to physical activity is seen in the study conducted by Putnam et al. (2003). Participants in the study linked their well-being (i.e., mental health, depression, and stress) directly to the inaccessibility of their build environment and its role as a barrier to physical activity. It is agreed upon that PwD are prone to secondary conditions including debilitating pain, depression, anxiety, stress and loneliness (Kinne et al., 2004; Olsen, 2018). The preventative interventions can, therefore, include not just participation in physical activities but also actively removing barriers to participation.

## Methods

This study aimed to generate evidence on the impact of Covid-19 restrictions on people with disabilities particularly pertaining to physical activity and associated issues. Inclusion criteria were any person with a disability in the Greater Glasgow and Ontario regions of Scotland and Canada, respectively. The study used process consent which assumes that consent is a process meaning participants can withdraw consent for their information to be used at any stage (Dewing, 2007).

The research formed part of a wider Canadian funded Social Science Humanities Research Council, study on parasport and disability sport and physical activity participation, as a legacy of mega-parasport events in Scotland and Canada. Participants already registered on the Project Echo online web research forum were invited to take part in an online focus group via Zoom. Additionally, members of partner organizations including Scottish Disability Sport (SDS) were invited to attend other focus groups. The study was wholly qualitative as we were interested in the meaning and impact that Covid-19 had, had on people's lives. Interviews and focus groups allowed for more discussion and sharing of experiences between participants. Which in turn helped the individuals participating, given some of the key areas centered on loneliness, isolation and mental and physical health and accessibility. It also created new networks for some of the participants, both in their home countries and internationally.

### Data Collection

Six focus groups were held using Zoom with participants signing up via an event created on the Project Echo forum website. These were advertised through Project Echo's social media platforms and through communication with Disability sports and advocacy organizations in Canada and Scotland. Additionally, invitations were sent directly to those people already registered on the Project Echo forum.

A total of 24 participants took part in the focus groups; seven of which were members of staff within disability sports organizations, six were performance pathway/ elite para athletes, five were competitive athletes at a non-performance pathway level and six were recreational or casual participants in sport or physical activity (see Table 1). Inclusion criteria for participants outlined that all those taking part in the focus groups were either a PwD or were employed in a management or policy role that works on the provision of sport and physical activity for PwDs. The focus group with staff were regional managers and the Senior Program Manager employed by a non-departmental public body Disability sport organization; SDS. They are directly responsible for implementing government policy and in influencing government policy through their advocacy role. The inclusion of this group was therefore deemed important to understand the evidence base they were using for policy decisions around coivd-19 and sport participation for PWD. Participants were not excluded on the basis of their level of activity with a broad range of experiences and participation levels sought to gain a broader understanding of the impact of Covid-19.

**Table 1:** Participant names and physical activity levels.

**Participant number**	**Pseudonym**	**Participant type**
P1	Jamie	Recreational
P2	Gareth	Recreational
P3	Angela	Recreational
P4	Anna	Recreational
P5	Sean	Casual
P6	Kai	Casual
P7	Mark	Competitive
P8	Ryan	Competitive
P9	Grace	Performance
P10	Chloe	Competitive
P11	Catherine	Competitive
P12	Alison	Competitive
P13	Hazel	Organizational
P14	Laura	Organizational
P15	Nick	Organizational
P16	Gerard	Organizational
P17	Amy	Organizational
P18	Karen	Organizational
P19	Leanne	Organizational
P20	Helen	Performance
P21	Grace	Performance
P22	Roy	Performance
P23	Richard	Performance
P24	David	Performance

In order to maximize accessibility in participation, the key focus group questions were emailed to participants prior to the session. Questions were provided on a Microsoft forms document which enabled participants to send answers in advance of the focus group which was seen as particularly useful for those with speech issues or whose disability caused them issues that meant they would be slow to type. Additionally, participants were encouraged to keep cameras on for those who may lip read, closed captioning was provided and use of the chat function was encouraged.

The key questions asked participants about their physical activity levels prior to the Covid-19 lockdown, how the lockdown and restrictions have impacted levels of physical activity, what has been a source of help in taking part in home-based exercises. Questioning also focused on how restrictions may have affected participants in other ways; socially, economically, and culturally as well as how participants felt about the easing of restrictions and beginning to access sport and physical activity again.

Focus groups were recorded using zoom functionality and transcribed verbatim. Each transcript of audio files and chats were analyzed by two members of the research team independently using a thematic analysis as a framework to guide the structure of researcher led analysis prior to discussion and agreement of key themes.

## Results and Discussion

Following analysis, four key themes have been identified throughout the focus group discussions; creativity, mental well-being, safety, and the exacerbation of disablement. Overall, it was evident from participants that Covid-19 restrictions have placed many different stresses and strains on the lives of PwD, however, despite this, the majority of participants attempted to make the best of the situation and adapt to new ways of working, socializing, and exercising.

### Creativity

A key theme emerging from the research was that of creativity. Adaptation to a new “normal” has become a key component of Covid-19 restrictions for most people with a substantial decrease in excursions outside of the home for reasons other than essential trips. The pandemic has created an environment that heightened self-isolation by reducing physical activity levels (Pinto et al., 2020). It was evident during focus groups that participants had been working on finding new and innovative ways to remain physically active. Using household items such as tins of food as weights and a wall to practice foot movement and shots for racquet sports including tennis and badminton formed were just some examples used by participants. Others also purchased exercise equipment and followed online workouts. Angela has been able to continue with some activity due to living in a rural and reasonably isolated area and has therefore felt comfortable going for runs, however, uneven terrain makes this a safety concern the longer lockdown continues with daylight beginning to fade in the evening.

Interestingly, there was a change in the mindset of some participants to find the positives of lockdown with one suggesting that she “felt like it was my winter training season indoors” (Catherine) and adapted her training schedule to fit accordingly. From a non-sporting perspective, Chloe found that whilst previously struggling to stay in contact with friends from university, her level of social contact with them has increased and they now stay in contact regularly via Zoom, “we said we can't believe we didn't do this before.” This proved an issue for Helen who lived in a very remote area with limited access to strong internet for phone signal which led to the opposite experience to that of Chloe as she felt more isolated from her training squad. She did manage to overcome this through perseverance in finding appropriate places where the signal was the strongest and this helped with both the social aspect of online contact but also in relation to her training as this allowed her to set up a gym in a barn building to take part in online workouts.

Richard outlined that his sport required very specialist equipment which made it difficult to adapt training, however, in order to maintain a social element amongst the club members, “as all clubs were shut down, but what we did manage to do was use an online game…. which kept the club spirit alive and there was a lot of tactics involved.” This allowed clubs to run virtual leagues for a social outlet as well as providing athletes the opportunity to work on their tactical knowledge of the sport.

Ryan has found other ways to stay busy and spends most evenings talking on the phone with two older people in his neighborhood who are isolated. It is therefore clear that participants are trying to make the best of the situations they currently find themselves in and are finding creative ways to maintain their levels of activity and also to use their time effectively. Those participants who appeared to manage to maintain their social relationships or indeed grow them during the pandemic, such as Ryan's developing friendship with the two older gentlemen and the online forums and sessions that the youth athletes took part in through their organizations all exhibited a lack of pessimism in comparison to other participants. The ability to “help others out with shopping and things helps me with my own anxiety and depression” (Kai) has aided the development of a more positive outlook on leaving the house during and post-lockdown.

A lack of social relationships may exacerbate feelings of isolation and loneliness which Hawkley and Cacioppo (2010) link directly to decreases in self-esteem and increases in pessimism. The associated overly cautious behaviors linked with pessimism were exhibited by several participants. Those who did not talk about having social contact with group sessions or conversations with friends expressed more fear and caution over moving out of lockdown restrictions, however, this may also be linked to previous experiences. Gareth talks about how he fears attitudes of people in the street who often “don't move to the side and bump into people” making social distancing very difficult and that his “worry is, society will be more exclusive” (Gareth). The vulnerability of health conditions makes it a very uncertain transition to post-lockdown with concerns over going out as it “depends on whether we get a vaccine” (Jamie) and that “they don't understand the practicality for people like me” (Jamie) of going into coffee shops and restaurants where people have touched many things or where people are wearing face coverings making lip reading very difficult.

However, whilst creativity was evident, it was clear that lockdown was continuing longer than anticipated and that the novelty of finding new training methods was becoming tedious with both Ryan and Mark suggesting that “hitting a ball against a wall is boring” and Angela suggesting that “I don't really enjoy exercising at home (kind of boring).”

It is not only those in lockdown that have been adapting, organizations have encountered the challenges of no longer being able to deliver sports sessions or have face to face contact with their athletes or people they support. The online content delivered by numerous organizations has been well-received by participants with there “being a different workout everyday” (Grace) available. Providing group sessions to allow athletes to maintain contact with their training groups has been important and beneficial to athletes, however, this is something that hasn't been as readily available for recreational or casual activity participants with support often being more specific to athletes on performance pathways. As restrictions begin to ease and some sports begin their return to play protocols, support staff are now experiencing difficulties with engagement in their online events “numbers are lower now because some of the guys are back out training” (Nick). Throughout Covid-19 restrictions, staff within SDS have been heavily involved in engaging with athletes from daily or weekly activity sessions online, weekly training plans and “keeping contact through weekly Zoom calls has helped maintain connectivity with (athletes)” (Laura). Amy further added that she has “offered phone calls/ video chats to individual athletes” and has encouraged family members to become involved in activities as a additional support mechanisms to athletes being able to maintain physical activity levels but this has been challenging due to them as staff members facing the same challenges of adapting to new work environments imposed by lockdown.

### Mental Well-Being

Physical activity has been positively associated with greater mental health and well-being (Bize et al., 2007; White et al., 2017) in part due to the positive social environment linked with participating in physical activity or sport, however, Tough et al. (2017) have highlighted that people with disabilities may have fewer opportunities to partake in such social gatherings. During the current pandemic, this enforced isolation from these physical and social settings has exacerbated the gap between people with disabilities and those with no disability. A general consensus emerged from the findings that indicated participants had found the lack of social contact “tough” (Anna) with different factors contributing to this. Chloe indicated a level of fear as, despite being vulnerable, she was the “least vulnerable in her family” therefore leaving the weekly trip to the supermarket to her. However, this element of fear and feeling unsafe will be explored further in later themes.

The words “Anxiety” and “depression” were commonly used throughout the focus groups creating emotive discussions resulting in Mark highlighting “having suicidal thoughts at various points because of the feelings of isolation and anxiety,” however, he has also been able to alter these feelings into more positive thoughts and this has been helped further by being able to return to sport due to being involved in an individual sport where social distancing is easily maintained “I used to feel suicidal myself, but I've just realized there's nothing you can really do about it and you need to step up a bit and say that it's better than being not here.” Anxiety and depression were terms also used by Anna and Gareth who both linked these to emotions of fear and isolation which again have been exacerbated by Covid-19 restrictions. Gareth admitted to feeling “anxiety because of Covid” and “finding some days harder than others” and feels “like I am missing out” by not being able to go out and go to work and see people he would normally see.

It is therefore evident that the removal of opportunities to safely navigate society and remain physically active has had a negative impact on the mental well-being of PwD. The uncertain times and potential of a second wave or lockdown of Covid-19 increases the urgency of understanding how to support PwD experiencing feelings of anxiety, depression, or isolation. Linked closely with the positive mindset previously mentioned in relation to creativity, David “firmly believe that athletes will come out stronger, both physically, and mentally when they realize that they can be adaptive when forced into a corner to make those changes.”

### Safety

For many, the lockdown measures have resulted in the closure of recreational facilities. Therefore, the only options are to engage in physical activities in one's own home or at outdoor public facilities like open fields, parks, and lakes. A common theme that emerged from the participants, was the concept of safety, or a lack thereof. Rimmer et al. (2004) identify emotional and psychological barriers as well as built and natural environment barriers as contributors to perceived safety issues amongst PwD. The concept of feeling unsafe was used by participants to describe a loss of protection from physical harm as well as a fear of being in a situation they could not control. For Angela, the feeling of not being “safe” was associated with the lack of hygienic accessible restrooms at outdoor facilities, particularly because easy access to restrooms was vital to the management of her coexisting condition. Increasing access to accessible and hygienic facilities becomes particularly more important during lockdown periods, as a lack of access has been shown to exacerbate loneliness and stress (Olsen, 2018).

For Jamie, the feeling of being unsafe in the outdoor built environment, existed even before the Covid-19 lockdown.

“For the past 5 years I have been terrified to go out, and I realized I need to get some exercise so obviously I tried to build up my confidence because I have mobility issues. The society is not that safe out there anymore for people with disabilities and I think it really impacted on me because of the potholes, I fell into a pothole, the pavements are not that flat, and it's very difficult to go out for a walk, ideally where you can walk safely and stuff.” (Jamie)

Studies have shown that the lack of features in the built environment can become facilitators or barriers to physical activity (Rosenberg et al., 2013; Misener et al., 2015; Eisenberg et al., 2017). Feelings of being unsafe have been exacerbated by the reduced access to hygienic and accessible facilities, including “safe accessible public transportation,” (Jamie) to outdoor facilities.

Angela admitted that the physical and environmental barriers made her “feel nervous,” thus showing a manifestation of biopsychosocial barriers to participation. Gareth and Kai also expressed anxiety in association with not feeling safe. Gareth was afraid that he would start experiencing pain, a symptom of his coexisting condition, while he was outdoors. He shared that “I really need to say to myself, today you need to go out, you will be fine.” Kai, expressed feeling anxiety because she did not feel safe neither in her residence nor outside of her residence. Kai shares a public kitchen with other people in her university residents. She concluded that since her disability is invisible, people around her did not respect the fact, or did not know that she was shielding. She found the behavior of her fellow residents irresponsible and dangerous. Their lack of social distancing and “stay at home” orders, left her feeling anxious about using the kitchen. Unfortunately, she felt like she had no option but to access food from outside, as she felt safer outside than inside.

Staff within SDS have recognized that their members and the athletes they support have faced multiple challenges from a mental well-being perspective with “many now experiencing poor mental health” (Laura) with many experiencing “isolation and change of routine due to shielding/activities stopping; becoming overwhelmed” (Amy). This social and psychological support provision adds to the role of the staff and has increased the importance of their role and it became clear that this contact also helped staff deal with the changes and struggles they were facing as “planned weekly activities, training and events have helped us all” (Karen).

### Exacerbation of Disablement

Gareth and Jamie raised important points about the considerations that need to be made regarding facemasks. Jamie's concern was that many people need to see her lips to help them understand what she is saying, and so wearing a mask would make things even more difficult for her. Jamie mentioned that she had ordered face visors, but her concerns were not only around her speech, but also her mobility. She feared that going to restaurants now would be tricky as she is prone to bumping into things. She was concerned that the things she bumped into might not be sanitized, also that they would need to be sanitized after she touches them, thus increasing work for the employees of the restaurant. Gareth added that this extra effort for people with mobility issues would further emphasize their perceived disability in public. He also mentioned that not wearing a facemask, when masks are becoming the norm, will soon be considered “antisocial.” Therefore, PwD that cannot wear masks are at risk of further social exclusion, thus perpetuating the “deviant” identity imposed by society (Low, 1996, p. 236).

Anna and Gemma, also noticed an increased need to assert physical distancing from people without a disability, especially since they use a wheelchair. Before lockdown, Gemma was not too bothered by people without a disability using the accessibility ramps, she would even wait for them to walk to the end before she got on. Recently, Gemma has noticed how her patience for this behavior has waned. Anna also noticed that getting on elevators meant that people without disabilities had to be more accommodating than usual. This sometimes meant missing an elevator because there was not enough space to physical distance. This change in behavior has the potential to undo the shedding of the deviant identity i.e., the ability to pass as less disabled. People in wheelchairs need to demand more physical distance than people without a disability, further highlighting their disability. As we move toward returning to physical sport, this has also led to further implications for clubs. Some sports clubs are able to re-open with increased physical distancing but trying to accommodate wheelchairs or the needs of PWD will take longer as sometimes, for example, there is not enough room on the athletics track, to ensure the safety of PwD and other athletes. This is fine if there is dedicated time for PwD but this is not always the case, thus the return to participation in sport for those PwD will take longer than able bodied athletes.

Scottish Disability Sport have worked with Scottish Young People's panel who participate in sport and Young Start Team panel aims to get athletes with a disability into coaching and look at career progression and we spoke with them about the changes in direction of funding and the policy shift in terms of what is now seen as a priority. SDS were trying to use their panels to help feed back to Scottish Government in terms of access to sport participation, put daily activities online and ensure they avert isolation and reduced income earning potential for athletes. They were trying to address this exacerbation of disablement. The dual role of this organization in supporting those with a disability to participate in sport but simultaneously respond to government and UN level policy in their advocacy role means they are campaigning to have their voice heard and the rights of athletes with a disability protected as well as provide a daily contact for PWD involved in sport.

## Implications and Conclusion

The UN (2020) outlines that there should be a collaborative approach between governments, health, and social care services, schools, and organizations representing social groups in order to promote and support physical activity at home. Where possible this should involve facilitating online resources, however, “low-tech and no-tech solutions must also be sought for those who currently lack access to the internet. Creating a flexible but consistent daily routine including physical exercise every day to help with stress and restlessness is advisable” UN (2020). This research confirms the UN's recommendations, by demonstrating the need for governments and agencies to provide affordable and accessible technology that enables continued contact with social networks. People tend to get resourceful and creative with technology, as they adapt to their new circumstances, but to ward off boredom there needs to be constant improvement of the resource. This is particularly true for those PwD at elite level sport, as they require dynamic physical activity.

Concerned parties must therefore, continue to monitor the needs of PwD and the current technologies ability to meet those needs. Numerous organizations have already started to include a physical activity component, as well as a socializing component, to their online platforms. This research shows that more time and resources need to be invested in augmenting these online features for PwD. Furthermore, these initiatives need to link to central/state government policy as UN (2020) argues for a collaborative approach between key agencies.

Not only are their physical activity levels reduced for elite athletes, but so are their sponsorship potentials, potential records and future livelihoods. This is unlikely to be resolved in the short term so there needs to be a response to helping elite para athletes access training facilities, in the same manner as able bodied have been afforded.

Safety remains a major concern for PwD. Both physical and psychosocial precipitants for not feeling safe, need to be addressed when fashioning initiatives to maintain or increase physical activity levels during lockdowns. This requires policy makers to consider how Covid-19 restrictions can further alienate PwDs by drawing more attention to their disability. Perhaps requiring service providers to wear visors or transparent masks, is a consideration policy makers and organizations need to entertain. Maintaining or increasing accessible, hygienic facilities should be enforced by governments, particularly at outdoor facilities. The need to ensure the rights of PwD to have continued access to physical activity is important, as without this, the evidence from our research has shown this is leading to increased mental health issues, a reduction in participation in physical activity and sport performance for those at elite level sport. Given we will be experiencing some form of physical distancing for a while and our research has shown similar results in Scotland and Canada we recommend there should be a collaborative approach between governments, international agencies and those responsible for the human rights and well-being of people with a disability to adopt an inclusive approach to access for physical activity and sport not just financially for shielding but to aid mental and physical health.

The impact of covid-19 for PwD, who are more at risk of further illness through reduced physical activity as evidenced by the WHO (2020) and our research, suggests the need for specific policy responses to help them maintain physical activity and exercise outdoors as well as indoors with others to reduce isolation. Policy makers, governing bodies, disability groups are all trying but at present those measures are not joined up, a collaborative approach will aid longer term solutions. Research shows that a reduction in physical activity can lead to increased levels of loneliness and mental distress. Our research then goes on to show that Covid-19 lockdowns and restrictions are also causing feelings of loneliness and mental distress which impact one's ability to be physically active, thus creating a cycle. Approaches to improving governments' Covid-19 response for PwD should therefore tackle the cycle by addressing biopsychosocial barriers to physical activity. Policy makers and agencies should ensure that PwD have access to and feel safe accessing physical activity alternatives like online solutions or outdoor facilities. Further research needs to be conducted on the iterative use and development of technology that mitigates barriers to physical activity. More research also needs to be conducted on how PwD are navigating new public health recommendations (and their subsequent exacerbation of disability), in a desire to remain physically active.

## Data Availability Statement

The datasets presented in this article are not readily available because as the data was recorded by video and audio the participants are identifiable and therefore only available to the approved research team. Requests to access the datasets should be directed to GM, gayle.mcpherson@uws.ac.uk.

## Ethics Statement

The studies involving human participants were reviewed and approved by University of Western Ontario. The patients/ participants provided their written and verbal informed consent to participate in this study.

## Author Contributions

The idea and conceptual framework for the article was that of GM and LM. The design of the fieldwork was GM and LC. LC, GM, and DK conducted the fieldwork. DK conducted the literature review with input from GM and LM on the theoretical model. LC led the analysis with input from GM, LM, and DK. GM and LC wrote the impact and conclusions. All authors contributed to the article and approved the submitted version.

## Conflict of Interest

The authors declare that the research was conducted in the absence of any commercial or financial relationships that could be construed as a potential conflict of interest.

## Footnotes

^1^We recognize the political nature and contested language for disabled persons/people with disabilities. We use the United Nation's accepted terminology of Persons with Disabilities (2006).
